# 

**Published:** 1902-12

**Authors:** 


### The Austin District Medical Society.

The Austin District Medical Society will meet in Austin, in semi-annual session, December 23rd inst. A fine program will be "pulled off," and a finer banquet will be pulled on, at the Driskill. Toasts to music, which will rise with voluptuous swell, and soft eyes will look lovingly on—from the balcony—I understand. Visitors from the West Texas, the Brazos Valley and other sister societies will be our guests. A general invitation to society members and those wishing to join the A. D. is extended.

